# The environmental impact of routine laboratory testing in maintenance haemodialysis

**DOI:** 10.1093/ckj/sfag113

**Published:** 2026-04-17

**Authors:** Maya Tickell-Painter, Alexander Woywodt, Callum Goolden, Robert J Shorten

**Affiliations:** Department of Pathology, Lancashire Teaching Hospitals NHS Foundation Trust, Preston, UK; Department of Nephrology, Lancashire Teaching Hospitals NHS Foundation Trust, Preston, UK; UK Health Security Agency; Department of Virology, Manchester Medical Microbiology Partnership; Department of Pathology, Lancashire Teaching Hospitals NHS Foundation Trust, Preston, UK; Division of Medical Education, University of Manchester

To the Editor,

Nephrologists are increasingly cognisant of resource usage and waste generation, particularly through the use of haemodialysis [1]. Patients receiving maintenance in-centre haemodialysis (ICHD) undergo regular laboratory testing. Such monitoring is typically done monthly, however less frequent testing has been proposed [2]. The environmental footprint of these tests has received very little attention so far.

We studied the environmental impact of ICHD routine blood tests at the haemodialysis unit at Chorley and South Ribble Hospital. We established the process flow for each test within the local policy for routine testing, including sampling, transport, and laboratory analysis. All blood tests and MRSA (methicillin resistant *Staphylococcus aureus)* swabs are taken at unit are transferred to our in-house laboratory, 14 miles away.

Carbon footprint analysis was performed using PAS 2050:2011 methodology. Study boundaries included greenhouse gas emissions relating to the production, transport, processing, and disposal of each consumable involved in laboratory testing and its associated packaging. The primary outcome measure is carbon dioxide equivalents (gCO_2_e).

We excluded processes where energy consumption does not vary with laboratory testing volumes, including heating, ventilation, air conditioning (HVAC), refrigeration, and processing of samples within automated systems. We excluded consumables already used in haemodialysis vascular access (e.g. gloves, sterile wipes for venepuncture). Identification and susceptibility testing of any potential MRSA isolates were excluded. Blood samples for virology testing followed the process flow for clinical biochemistry.

**Figure 1:**
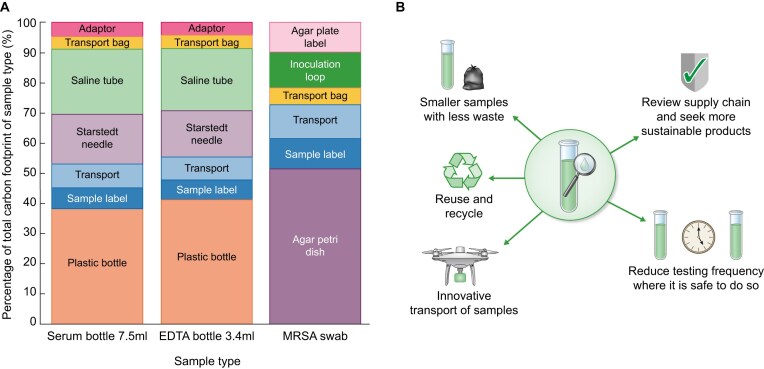
(A) Sources of CO_2_e emissions for each sample type. (B) Potential routes to reduce emissions from regular laboratory testing.

The carbon footprint of each sample type was: Ochre top (7.5 ml) bottle 117gCO_2_e, EDTA bottle (3.4 ml) 125gCO_2_e and MRSA swab 110gCO_2_e. For each patient, the carbon footprint of the annual blood tests collected is 4438gCO_2_e. MRSA swab processing contributed an additional 440gCO_2_e. The combined total is equivalent to travelling 18 miles in a medium petrol car. For blood tests, the most significant contribution to the CO_2_e emissions was the plastic tube used for venepuncture, whilst for MRSA swabs, this was the Agar petri dish (Fig. 1A).

Each year 82 patients attend this haemodialysis unit. We calculate that the total annual carbon footprint related to all laboratory tests performed on these patients is 399,996gC0_2_e, which is equivalent to travelling 1476 miles in a medium petrol car.

Plastic consumables, particularly blood collection tubes, account for the majority of the carbon footprint. Strategies to reduce the environmental impact of routine testing should consider alternative materials, improved recycling, and more sustainable supply chains. However, as individual dialysis units have limited control over these variables, reducing testing frequency may be more feasible. If we adjusted our testing schedule to that proposed by Chidiac *et al*. [2], the annual carbon saving would be 1337gCO_2_e per patient.

Existing literature on the environmental harms of laboratory testing is limited, and inter-centre comparisons are challenging [3]. Our figures differ slightly from other published literature. McAllister *et al*. [4] estimated that the carbon footprint of a full blood count examination was 116gCO_2_e (125gCO_2_e in our paper), and a urea and electrolytes test was 99gCO_2_e (vs. 117gCO_2_e). This may be due to different study boundaries, laboratory processes, and emission factors in a different country.

This study identifies routine laboratory testing as a meaningful contributor to the environmental footprint of haemodialysis care. We have previously highlighted that areas other than dialysis, such as the commute to dialysis treatments and appointments, also have an environmental footprint [5]. We suggest that nephrologists consider targeted reductions in testing frequency where this is safe [2]. Smaller sample sizes, innovation in consumables, and innovative approaches to sample transport may also help (Fig. 1B). As healthcare systems face mounting pressure to meet ambitious climate targets, embedding environmental considerations into routine clinical decision-making will be essential to delivering sustainable, high-quality care.
